# Structural Insights into the Affinity of Cel7A Carbohydrate-binding Module for Lignin*

**DOI:** 10.1074/jbc.M115.673467

**Published:** 2015-07-24

**Authors:** Kathryn L. Strobel, Katherine A. Pfeiffer, Harvey W. Blanch, Douglas S. Clark

**Affiliations:** From the Department of Chemical and Biomolecular Engineering, University of California Berkeley, Berkeley, California 94720

**Keywords:** biofuel, carbohydrate-binding protein, cellobiohydrolase, cellulase, protein engineering, lignin

## Abstract

The high cost of hydrolytic enzymes impedes the commercial production of lignocellulosic biofuels. High enzyme loadings are required in part due to their non-productive adsorption to lignin, a major component of biomass. Despite numerous studies documenting cellulase adsorption to lignin, few attempts have been made to engineer enzymes to reduce lignin binding. In this work, we used alanine-scanning mutagenesis to elucidate the structural basis for the lignin affinity of *Trichoderma reesei* Cel7A carbohydrate binding module (CBM). *T. reesei* Cel7A CBM mutants were produced with a *Talaromyces emersonii* Cel7A catalytic domain and screened for their binding to cellulose and lignin. Mutation of aromatic and polar residues on the planar face of the CBM greatly decreased binding to both cellulose and lignin, supporting the hypothesis that the cellulose-binding face is also responsible for lignin affinity. Cellulose and lignin affinity of the 31 mutants were highly correlated, although several mutants displayed selective reductions in lignin or cellulose affinity. Four mutants with increased cellulose selectivity (Q2A, H4A, V18A, and P30A) did not exhibit improved hydrolysis of cellulose in the presence of lignin. Further reduction in lignin affinity while maintaining a high level of cellulose affinity is thus necessary to generate an enzyme with improved hydrolysis capability. This work provides insights into the structural underpinnings of lignin affinity, identifies residues amenable to mutation without compromising cellulose affinity, and informs engineering strategies for family one CBMs.

## Teaser

**Background:** Non-productive adsorption to lignin hinders cellulase efficiency.

**Results:** 31 alanine mutants of the carbohydrate-binding module (CBM) exhibit highly correlated cellulose and lignin affinity.

**Conclusion:** The cellulose-binding face of CBM is responsible for the majority of lignin affinity, although four mutations were identified that selectivity decrease lignin adsorption.

**Significance:** The mutations studied inform future engineering efforts of fungal CBMs.

## Introduction

Lignocellulosic biomass is an abundant, low-cost resource for the renewable production of fuels and chemicals. Unfortunately, lignocellulose is highly resistant to enzymatic degradation, necessitating high enzyme loadings that raise the cost of second-generation biofuels (1). The recalcitrance of biomass stems in part from the presence of lignin. Lignin, a major component of lignocellulosic biomass, inhibits the efficiency and recyclability of cellulase enzymes. Numerous studies have found that removing lignin from biomass increases the final hydrolysis yield (*e.g.* Refs. 2–7), and adding supplemental lignin decreases the final hydrolysis yield (*e.g.* Refs. 8–14). Lignin impedes hydrolysis through two main mechanisms: physically blocking cellulose access (5, 6) and non-productively binding enzymes (15), thus lowering the effective enzyme concentration. Cellulase adsorption to lignin has been well documented for both cellulase mixtures (10, 16) and individual enzymes (9, 17, 18), although the structural basis of the interaction is not fully elucidated.

Most fungal cellulases consist of two domains: a catalytic domain (CD)^2^ and a carbohydrate-binding module (CBM) connected by a highly glycosylated, flexible linker (19). The CBM is responsible for increasing the effective concentration of the catalytic domain on the surface of its substrate (20). Fungal CBMs are highly conserved, 30–40 amino acid domains with a planar cellulose-binding surface. Three aromatic amino acids, arranged such that they align with a sequence of every other glucose unit on the surface of cellulose, are crucial for cellulose binding (21). Polar amino acids on the same face of the CBM are also important for binding to cellulose, presumably through the formation of hydrogen bonds (21). Along with binding to cellulose, the CBM is also responsible for the majority of lignin affinity. Fungal cellulases expressed without the CBM, or with the CBM removed, exhibit greatly reduced lignin affinity (17, 22, 23), highlighting the role of the CBM in lignin adsorption.

Studies of CBM lignin affinity have implicated hydrophobic and electrostatic interaction mechanisms. In a comparison of *Trichoderma reesei* Cel7A and Cel7B, Borjesson *et al.* (24) showed that Cel7B had higher affinity to lignin, perhaps due to the more hydrophobic surface on the flat face of the CBM together with an additional exposed aromatic residue on the rough face. Sammond *et al.* (18) found that the adsorption of enzymes to lignin surfaces correlates with solvent-exposed hydrophobic clusters. Increasing the carboxylic acid content, and therefore negative charge, of lignin has been shown to decrease the non-productive binding of cellulases and increase the enzymatic hydrolysis of cellulose (12), presumably due to increased repulsive electrostatic interactions between the enzymes and lignin. Adding negative charge to cellulases by succinylation has also been shown to reduce lignin inhibition (25). Improved hydrolysis of cellulose in the presence of lignin at high pH, along with reduced lignin binding at high pH, also demonstrates the importance of electrostatic interactions (26).

Despite the numerous studies documenting cellulase adsorption to lignin, the structural basis has not been fully elucidated and few attempts have been made to engineer enzymes for reduced lignin affinity. Rahikainen *et al.* (26) showed that mutation of one aromatic amino acid on the flat face of the *T. reesei* Cel7A CBM from tyrosine to alanine reduced lignin affinity, whereas mutation from tyrosine to tryptophan increased lignin affinity. Interestingly, although the cellulose affinity of the alanine mutant was reduced to the level of the catalytic domain alone, the lignin affinity of the mutant remained greater than the catalytic domain, indicating that the mutated enzyme retained considerable lignin affinity.

In this work, we employed extensive alanine scanning mutagenesis of the entire *T. reesei* Cel7A CBM to identify residues involved in lignin adsorption and highlight locations where mutation may increase the selectivity for cellulose. Toward the overall goal of generating a lignin-resistant enzyme, we evaluated the most promising mutants for hydrolysis in the presence of lignin to investigate the extent to which lignin affinity must be reduced to decrease lignin inhibition.

## Experimental Procedures

### 

#### 

##### Materials

Microcrystalline cellulose PH-105 (Avicel), protease from *Streptomyces griseus,* 4-methylumbelliferyl β-d-cellobioside, Tween 20, cellulase from *T. reesei* (Celluclast), and papain were purchased from Sigma. Accelerace 1500 was a gift from DuPont. *Aspergillus niger* β-glucosidase was purchased from Megazyme. Acid-pretreated *Miscanthus* was produced by two-step dilute acid pretreatment on the pilot scale by Andritz, Glens Falls, NY. Solids were washed with water to neutral pH and lyophilized before lignin preparation.

##### Lignin Preparation

Lignin residues were isolated from acid-pretreated *Miscanthus* by enzymatic hydrolysis followed by *S. griseus* protease treatment to remove bound enzyme. Pretreated, rinsed, lyophilized biomass was ball milled for 5 min (Kleco ball mill, Visalia, CA). Enzymatic hydrolysis was carried out at 50 °C, pH 5.0, with 4 weight % biomass and 0.25 ml of Accelerase 1500/g of biomass. The reaction mixtures were centrifuged and the supernatant replaced with fresh buffer and enzyme (0.25 ml of Accelerase/g original biomass) every 24 h. After 7 days of hydrolysis, the solids were separated by centrifugation and washed with acidified water (pH adjusted to 3.5 with HCl to avoid lignin solubilization). Bound protein was removed by an overnight protease treatment at 37 °C, pH 7.5, with 2 weight % solids and 0.3 mg/ml of protease. The solids were then washed with acidified water and heated in a boiling water bath for 60 min to deactivate any remaining protease. The solids were extensively washed with acidified water and freeze-dried.

##### Lignin Analysis

Compositional analysis of pretreated biomass and lignin was performed as previously described, with sugar concentrations determined by HPLC (Dionex) (27). Protein content in the solid samples was determined by total nitrogen analysis. The analysis was carried out at the University of California Berkeley Microanalytical Facility using a PerkinElmer 2400 Series II combustion analyzer.

##### Purification of T. reesei Cel7A

Cel7A was purified from Celluclast using a Q-Sepharose anion exchange column followed by a MonoQ ion exchange column. Prior to injection to the MonoQ column, the crude Cel7A was treated with 0.1% Tween 20 to disrupt nonspecific interactions that were found to exist between Cel7A and Cel7B. Fractions from the MonoQ were checked for purity by SDS-PAGE and an assay for contaminant endoglucanase activity. Assays contained 2% carboxymethyl cellulose and roughly 10 μm enzyme in 100 mm sodium acetate, pH 4.5. Assays were incubated at 50 °C overnight and endoglucanase activity was assessed by adding 2,4-dinitrosalycilic acid, incubating at 90 °C for 5 min, and visually inspecting for the presence of brown color in the reactions. Fractions showing no endoglucanase activity were pooled and buffer exchanged into 50 mm sodium acetate, pH 5.0.

##### Isolation of the T. reesei CD

The *T. reesei* CD was cleaved from the linker and CBM using the protease papain. Papain was activated by incubating a 28 mg ml^−1^ solution with 2 mm dithiothreitol and 2 mm EDTA in 100 mm sodium phosphate, pH 7.0, for 30 min at room temperature. Cleavage reactions contained a (w/w) ratio of 1:100 papain to Cel7A in 50 mm sodium acetate, pH 5.0, and were incubated overnight at room temperature with stirring. The CD was purified from the protease and CBM using a Q-Sepharose anion exchange column.

##### Small-scale Enzyme Production

The *Talaromyces emersonii cel7A* CD or a fusion of *T. emersonii* Cel7A CD and the *T. reesei cel7A* linker and CBM was cloned into a high-copy number plasmid (pCu424 (28)) and produced in YVH10 *pmr1*Δ *Saccharomyces cerevisiae* (29). Mutations were made by site-directed mutagenesis (30). For screening all mutants, 20 ml of SC-Trp medium was inoculated with *S. cerevisiae* containing the *cel7A* gene and grown for 3 days at 30 °C, 220 rpm. Cultures were spun down at 5,000 × *g* for 5 min and resuspended in SC medium supplemented with 500 mm Cu_2_SO_4_, then cultured for protein production for 3 days at 25 °C, 220 rpm. Supernatant was collected and buffer exchanged into 50 mm sodium acetate, pH 5.0, using Amplicon spin concentrators (Millipore).

##### Large-scale Enzyme Production and Purification

For large-scale production, 1 liter of SC-Trp medium was inoculated with *S. cerevisiae* containing the *cel7A* gene and grown for 3 days at 30 °C, 220 rpm. Cultures were spun down at 5,000 × *g* for 5 min and resuspended in YPD medium supplemented with 500 mm Cu_2_SO_4_ and cultured for 3 days at 25 °C, 220 rpm. Enzyme purification was carried out using a two-step procedure beginning with anion exchange chromatography. Active fractions were combined and further purified by hydrophobic interaction chromatography. Pure fractions (single band via SDS-PAGE) were combined and buffer exchanged into 50 mm sodium acetate buffer, pH 5.0.

##### Enzyme Quantification

Purified enzymes were quantified by absorbance at 280 nm using the calculated molar extinction coefficient of 76,240 m^−1^ cm^−1^ for the fusion enzyme and 71,120 m^−1^ cm^−1^ for the catalytic domain. Unpurified enzyme and enzyme remaining in the supernatant after binding assays were quantified by their activity on a soluble substrate, 4-methylumbelliferyl β-d-cellobioside. The specific activity of 10.2 μmol of MU (μmol of enzyme/min)^−1^ was measured for the purified wild type, catalytic domain, and mutant chimeras. Assays were conducted in a total volume of 100 μl with 1 mm 4-methylumbelliferyl β-d-cellobioside, pH 5.0, at 45 °C for 8 min, followed by a denaturation step at 98 °C for 2 min. Sodium hydroxide was added to a final concentration of 0.1 m and the fluorescence was measured on a Paradigm plate reader (Beckman Coulter) with excitation of 360 nm and emission of 465 nm.

##### Avicel and Lignin Binding Screen

Unpurified, buffer-exchanged mutants were normalized to the same concentration of active Cel7A. Binding to Avicel and lignin was measured in 50 mm sodium acetate buffer, pH 5, using 15 mg/ml of Avicel or lignin and 125 nm enzyme. Experiments were conducted in a total volume of 70 μl in a rotating shaker (300 rpm) at room temperature. An initial time course with the wild type enzyme was measured to determine the time required for binding to reach equilibrium. Equilibrium was reached after ∼30 min. Subsequent assays were performed for 1 h. After equilibration, solids were separated by centrifugation and the supernatant was analyzed for free, active enzyme using 4-methylumbelliferyl β-d-cellobioside. The amount of bound enzyme was calculated from the difference between added enzyme and enzyme remaining in solution. The values plotted in Fig. 2 are bound enzyme divided by enzyme remaining in solution. At the low enzyme concentrations employed, these values approximate the partition coefficient, α, as defined in Equation 1. The values reported are averages of duplicate experiments and the errors are calculated as standard deviations.

The one-site Langmuir isotherm at low free protein concentrations can be written as,

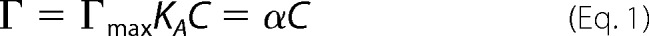
 where Γ is the amount of adsorbed protein, Γ_max_ is the amount of adsorbed protein at saturation, *K_A_* is the Langmuir affinity constant, *C* is the concentration of free protein, and α is the partition coefficient.

##### Adsorption Isotherms

Adsorption isotherm measurements were performed in 50 mm sodium acetate buffer, pH 5, using 10 mg/ml of Avicel or lignin and initial enzyme concentrations ranging from 0.015 to 4.0 μm. Experiments were conducted using the same conditions as the binding screen. A one-site Langmuir adsorption isotherm (Equation 2) was fit to the binding data by non-linear least squares optimization to determine *K_A_*, Γ_max_, and their corresponding standard deviations.

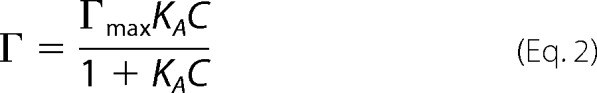


##### Hydrolysis of Avicel Supplemented with Lignin and Miscanthus

Hydrolysis reactions were performed with 10 mg/ml of Avicel, 10 mg/ml of Avicel supplemented with 10 mg/ml of isolated lignin, or 20 mg/mlof acid-pretreated *Miscanthus* in 50 mm sodium acetate buffer, pH 5, with 0.5 μm Cel7A in Avicel reactions and 1.0 μm Cel7A in *Miscanthus* reactions. Reactions were carried out in a thermally controlled incubator with end-over-end mixing at 60 °C for enzymes containing the *T. emersonii* catalytic domain and at 55 °C for enzymes containing the *T. reesei* catalytic domain. After 48 h, reaction mixtures were filtered and analyzed immediately for glucose by HPLC (Shimadzu). The values reported are means of duplicate samples and the errors are calculated as standard deviation.

##### Predicting the Amount of Enzyme Bound to Cellulose during Hydrolysis

Equations 3–5 were used to predict the amount of cellulose-bound enzyme in the presence of lignin.

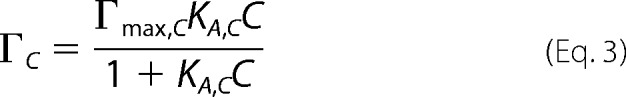


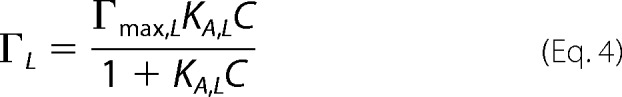


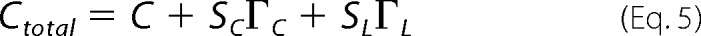
 where Γ*_C_* and Γ*_L_* are the amounts of enzyme bound per gram of solid to cellulose and lignin, respectively; *K_A,C_* and *K_A,L_* are the Langmuir binding constants to cellulose and lignin, respectively; Γ_max,_*_C_* and Γ_max,_*_L_* are the amounts of adsorbed protein at saturation to cellulose and lignin, respectively; *C* is the concentration of protein remaining free in solution; *C*_total_ is the initial protein concentration, and *S_C_* and *S_L_* are the concentrations of cellulose and lignin, respectively.

## Results

### 

#### 

##### Isolation and Characterization of Lignin from Pretreated Miscanthus

Lignin used in this study was isolated from acid-pretreated *Miscanthus* using a commercial cellulase mixture (Accelerase 1500, DuPont) to ensure that the lignin was as similar as possible to lignin present in an industrial hydrolysis reaction. Excess enzyme and long digestion times were used to remove all accessible carbohydrates. The solid residue was then treated with *S. griseus* protease to remove bound enzymes. The compositions of the starting biomass and resulting isolated lignin are presented in Table 1. The Klason lignin, remaining carbohydrates, and protein content are similar to protease-treated lignin used in previous studies (9–11, 17, 26).

**TABLE 1:** **Characterization of pretreated *Miscanthus* and isolated lignin**

	Glucan	Xylan	Arabinan	Klason lignin	Ash	Nitrogen
	%					
Acid-pretreated *Miscanthus*	55.6	0.54	0.11	33.6	9.73	<0.2
Isolated lignin	6.21	0.39	0.13	77.0	14.51	0.34

##### Lignin Inhibition of Cel7A and Isolated Catalytic Domain

**FIGURE 1.**
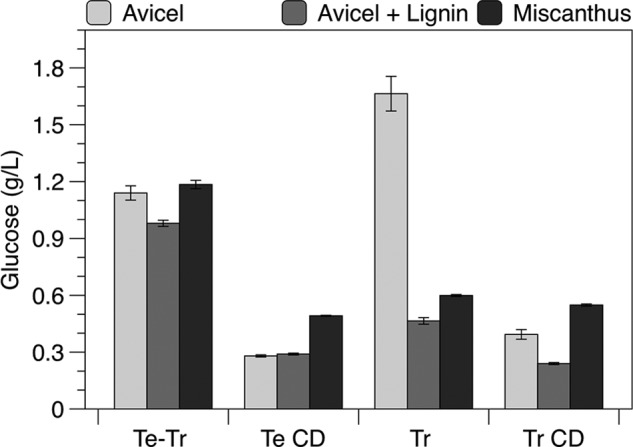
**Hydrolysis by *T. reesei* (*Tr*) and *T. emersonii* (*Te*) Cel7A species, including each CD, and the Te-Tr chimera.** Glucose produced after 48 h with 10 g/liter of Avicel, 10 g/liter of Avicel with 10 g/liters of lignin, or 20 g/liters of *Miscanthus*. All reactions were supplemented with *A. niger* β-glucosidase. Reactions were performed at 60 °C for *T. emersonii* (*Te*) enzymes and 55 °C for *T. reesei* (*Tr*) enzymes with 0.5 μm Cel7A in Avicel reactions and 1.0 μm Cel7A in *Miscanthus* reactions. Values presented are means of duplicate samples and errors are S.D.

We compared the lignin inhibition of natively expressed *T. reesei* Cel7A to that of the chimera used for engineering in this study, recombinantly expressed *T. emersonii* Cel7A catalytic domain fused to the *T. reesei* Cel7A linker and carbohydrate-binding module (Te-Tr chimera). Fig. 1 shows the hydrolysis of Avicel, Avicel in the presence of supplemental lignin, and acid-pretreated *Miscanthus* by each enzyme and the corresponding isolated CD.

The Te-Tr chimera was less inhibited by lignin than *T. reesei* Cel7A. The addition of lignin to Avicel decreased the glucose generated by ∼15% for the Te-Tr chimera, and 73% for the full-length *T. reesei* enzyme. The *T. emersonii* CD was not inhibited by supplemental lignin, indicating that the inhibition of the Te-Tr chimera is due entirely to the linker and CBM, our mutagenesis target. The *T. reesei* CD generated 38% less sugar in the presence of lignin. Although both CDs were less inhibited by lignin, they generated significantly less sugar than the corresponding full enzyme.

##### CBM Engineering Approach

We used alanine-scanning mutagenesis to identify residues that contribute to lignin affinity. The isolated *T. emersonii* catalytic domain, wild type Te-Tr chimera, and 31 Te-Tr mutant chimeras, each with one position in the CBM changed to alanine, were produced in *S. cerevisiae* strain YVH10 *pmr1*Δ. This strain includes an overexpressed protein-disulfide isomerase for increased Cel7A titers and a knock-out of the *PRM1* gene for reduced hyperglycosylation (29). Each residue in the CBM was mutated to alanine except cysteine residues involved in disulfide bonds and native alanine residues.

##### Binding Screen

The Cel7A mutants were secreted into defined medium, buffer exchanged, and screened for cellulose and lignin binding without purification. Binding was quantified by measuring the Cel7A activity remaining in the supernatant using a soluble substrate, 4-methylumbelliferyl β-d-cellobioside, which permitted specific detection of Cel7A among other secreted proteins. Each mutant was screened for binding at low enzyme concentration, in the range where the Langmuir isotherm is linear, permitting direct calculation of the partition coefficient from the amount of bound and free enzyme (Equation 1 under “Experimental Procedures”).

##### Impact of Point Mutations on CBM Adsorption to Avicel

**FIGURE 2.**
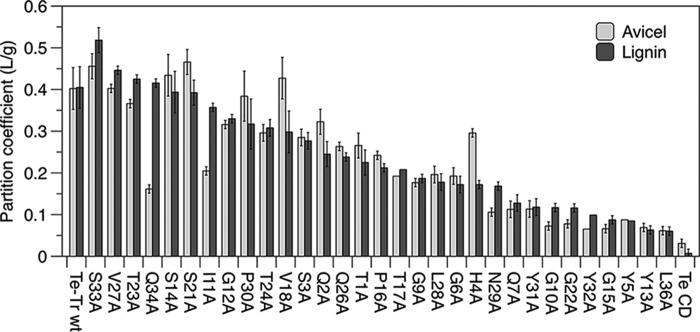
**Enzyme partition coefficients (α) for Avicel and lignin calculated from binding experiments at room temperature and an initial enzyme concentration of 125 nm.** Values presented are means of duplicate samples and errors are S.D.

**FIGURE 3.**
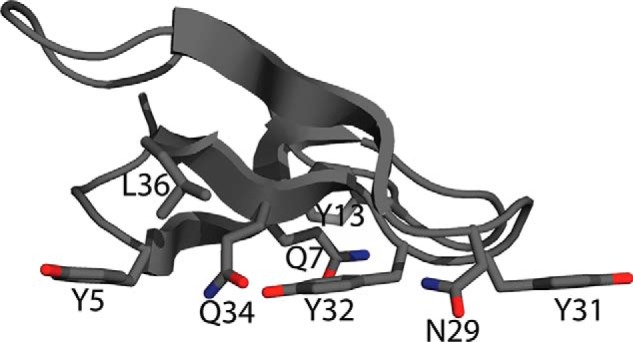
***T. reesei* Cel7A CBM structure showing side chains important to cellulose or lignin adsorption (38).**

The cellulose partition coefficient of each mutant is shown in Fig. 2 (*light bars*) and the CBM structure is shown in Fig. 3. The CD alone has significantly less affinity for cellulose than the chimera containing the wild type CBM, as expected. All mutants have an Avicel partition coefficient in the range between the wild type and CD alone. As previously reported, mutating the three tyrosines on the planar face of the CBM (Tyr-5, Tyr-31, and Tyr-32) as well as polar residues on the same face (Asn-29, Gln-34) to alanine significantly reduced cellulose binding (21). Mutation of Gln-7, Gly-10, Tyr-13, Gly-15, Gly-22, and Leu-36 to alanine also significantly reduced binding to cellulose (<30% of wild-type affinity).

##### Impact of Point Mutations on CBM Adsorption to Lignin

The lignin partition coefficient of each mutant is shown in Fig. 2 (*dark bars*). The *T. emersonii* CD has negligible lignin affinity when compared with the fusion containing the *T. reesei* linker and CBM. We found that mutation of Tyr-5, Gln-7, Gly-10, Tyr-13, Gly-15, Gly-22, Tyr-31, Tyr-32, and Leu-36 had the greatest impact on lignin affinity. Tyr-5, Gln-7, Tyr-31, and Tyr-32 are residues on the cellulose-binding face of the CBM, as seen in Fig. 3. Although these mutants had significantly reduced lignin affinity compared with the wild type chimera, all mutants retained much greater lignin affinity than the isolated CD.

##### Impact of Point Mutations on CBM Selectivity

**FIGURE 4.**
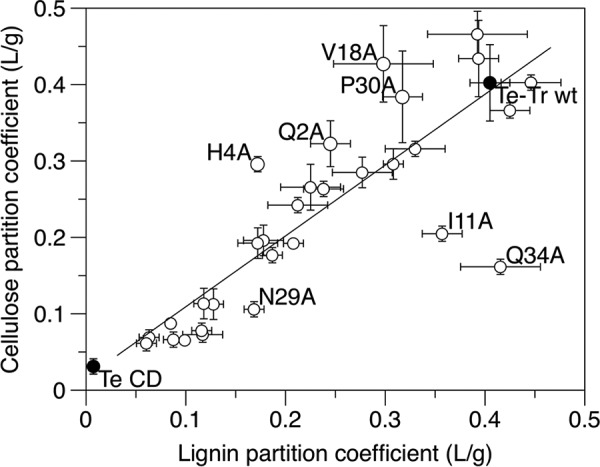
**Binding selectivity of Te-Tr wild type, CD, and all mutants, as evidenced by a scatter plot of partition coefficients.** Values presented are means of duplicate samples and errors are S.D.

For hydrolysis of lignocellulose, it is important to consider the selectivity of adsorption to cellulose over lignin. As seen in Fig. 4, lignin and cellulose affinity of the mutants were highly correlated (*R*^2^ = 0.92), although a few outliers exhibited a selective reduction in cellulose or lignin adsorption. Q34A, I11A, and N29A reduced cellulose selectivity, whereas Q2A, H4A, V18A, and P30A increased cellulose selectivity.

##### Adsorption Isotherms of Selected Mutants

**FIGURE 5.**
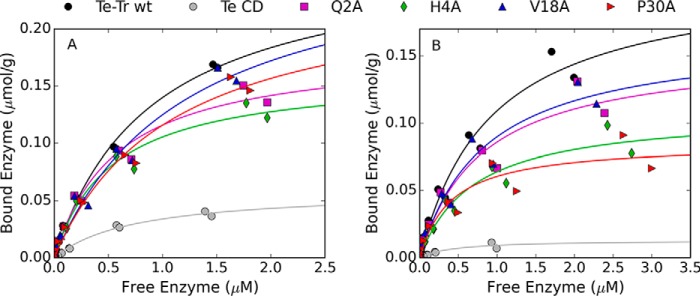
**Adsorption isotherms to Avicel (*A*) and lignin (*B*) measured at room temperature.** The *lines* show the fit to a one-site Langmuir binding isotherm.

The wild type enzyme, CD alone, and the alanine mutants with increased selectivity toward Avicel were characterized further. The partition coefficient calculated from the binding screen does not provide enough information to predict binding behavior at higher concentrations, where the Langmuir isotherm is no longer linear. Therefore, full Langmuir isotherms were measured and fit using non-linear least squares regression to determine the adsorption at saturation (Γ_max_) and the Langmuir adsorption constant (*K_A_*). The isotherms are shown in Fig. 5 and the model parameters are listed in Table 2.

**TABLE 2:** **Langmuir adsorption parameters**

	Avicel			Lignin		
	*K_A_**^a^*	Γ_max_	α	*K_A_*	Γ_max_	α
	μ*m*^−*1*^	μ*mol/g*	*liter/g*	μ*m*^−*1*^	μ*mol/g*	*liter/g*
Wild type	1.10 ± 0.11	0.27 ± 0.01	0.29 ± 0.03	1.07 ± 0.21	0.21 ± 0.02	0.23 ± 0.05
CD	1.34 ± 0.17	0.059 ± 0.003	0.08 ± 0.01	2.52 ± 0.77	0.013 ± 0.001	0.03 ± 0.01
Q2A	1.74 ± 0.27	0.18 ± 0.01	0.32 ± 0.05	1.26 ± 0.33	0.15 ± 0.02	0.19 ± 0.05
H4A	1.90 ± 0.26	0.16 ± 0.01	0.31 ± 0.05	1.46 ± 0.32	0.11 ± 0.01	0.16 ± 0.04
V18A	0.82 ± 0.21	0.28 ± 0.04	0.23 ± 0.06	1.19 ± 0.30	0.17 ± 0.02	0.20 ± 0.05
P30A	0.92 ± 0.17	0.24 ± 0.02	0.22 ± 0.05	2.49 ± 0.75	0.08 ± 0.01	0.21 ± 0.07

*^a^ K_A_*_,_ Langmuir adsorption constant; Γ_max_, adsorption at saturation; a, partition coefficient (a = *K_A_* Γ_max_) indicated with their standard deviation.

Qualitatively, the mutations decreased lignin adsorption more than Avicel adsorption, as seen in Fig. 5. The partition coefficients to Avicel derived from the Langmuir isotherms (α = *K_A_*Γ_max_) are similar for the wild type and mutant enzymes (Table 2), as expected based on data from the binding screen. The saturating amount of enzyme adsorbed to lignin, Γ_max_, is lower for each of the mutants than the wild type enzyme, although the affinity constants and partition coefficients are within error of the wild type enzyme.

##### Hydrolysis of Avicel in the Presence of Lignin and Acid-pretreated Miscanthus

**FIGURE 6.**
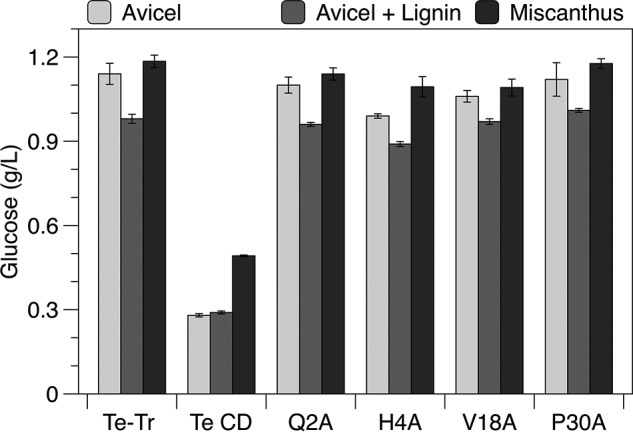
**Hydrolysis of Avicel with and without supplemental lignin and of acid-pretreated *Miscanthus*.** Glucose produced after 48 h with 10 g/liter of Avicel, 10 g/liter of Avicel with 10 g/liter of lignin, or 20 g/liter of *Miscanthus*. All reactions were supplemented with *A. niger* β-glucosidase. Reactions were performed at 60 °C with 0.5 μm Cel7A in Avicel reactions and 1.0 μm Cel7A in *Miscanthus* reactions. Values presented are means of duplicate samples and errors are S.D.

To examine the impact of increased cellulose specificity on hydrolysis in the presence of lignin, the wild type, CD, and mutant enzymes were tested for their ability to hydrolyze Avicel, Avicel with added lignin, and acid-pretreated *Miscanthus* (Fig. 6). Although each of the mutants displayed increased selectivity toward binding cellulose, none generated more glucose than the wild type enzyme.

##### Predicting the Amount of Enzyme Bound to Cellulose

We sought to explain the hydrolysis results and predict levels of binding selectivity necessary for hydrolytic improvement by using the Langmuir adsorption model. The first step of cellulose hydrolysis is adsorption to the surface. Predicting the amount of enzyme adsorbed to cellulose can therefore lend insight into the performance of enzymes with different affinities. Using the Langmuir parameters (*K_A_* and Γ_max_) shown in Table 2, the approximate amount of enzyme bound to Avicel under the hydrolysis conditions employed in this study was calculated for the wild type chimera, CD, and the four selected mutants. This prediction is restricted to hydrolysis of pure cellulose with supplemental lignin, due to the use of Langmuir parameters measured on the pure substances.

**FIGURE 7.**
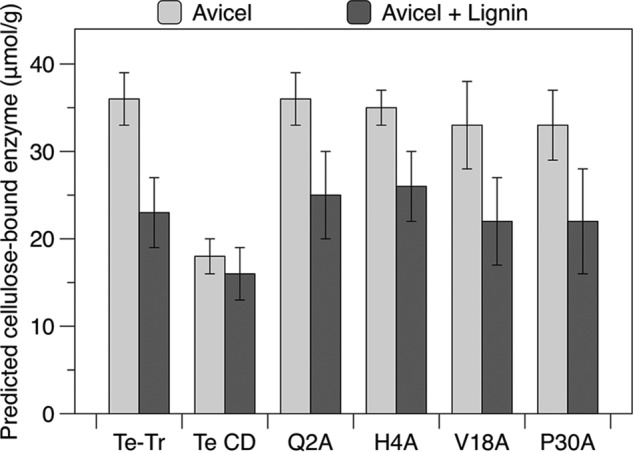
**Predicted amounts of enzyme bound to Avicel in the absence or presence of supplemental lignin.** Bound enzyme is calculated using the Langmuir parameters in Table 2 for a solution with 0.5 μm total enzyme and 10 g/liter of Avicel, with or without 10 g/liter of lignin. Error is presented as the range of values possible given the standard deviation in the Langmuir affinity constant (*K_A_*) and saturation adsorption constant (Γ_max_).

As shown in Fig. 7, none of the mutants are predicted to have significantly more enzyme bound to Avicel than the wild type, which explains why none of the mutants generated more glucose during the hydrolysis assays. The catalytic domain is predicted to bind in similar amounts to cellulose in the presence and absence of lignin, as expected given that it performed equally well in hydrolysis assays with and without lignin (Fig. 6). The full-length wild type enzyme and each of the mutants are predicted to have between 3 and 55% less enzyme bound to cellulose when lignin is present, which covers the range of lignin inhibition observed in the hydrolysis assays.

## Discussion

Non-productive adsorption of cellulases to lignin reduces the effective enzyme concentration during biomass hydrolysis, increasing the overall cost of producing biofuels. Although it is well known that the CBM drives this non-productive adsorption, the mechanisms of CBM lignin affinity have not been fully elucidated. We employed alanine-scanning mutagenesis of the *T. reesei* Cel7A CBM to reveal the structural basis of lignin affinity and identify residues where mutation increases the selectivity for cellulose over lignin. Our studies focused on *T. reesei* Cel7A because it is the most abundant enzyme in many commercial cellulase mixtures. Each CBM mutant was produced with a linker and CD to ensure realistic binding conditions and enable hydrolysis studies. We used the *T. reesei* Cel7A CBM and linker, and the *T. emersonii* Cel7A CD. This CD is homologous to *T. reesei*, and unlike the *T. reesei* enzyme, expresses well in yeast, making enzyme production and screening tractable.

Although the Te-Tr chimera was less inhibited by lignin than the native *T. reesei* enzyme, the inhibition of the chimera was entirely due to the CBM, making it a well suited construct for CBM engineering. Surprisingly, the *T. reesei* Cel7A CD was also inhibited by supplemental lignin. This result is unexpected given the general consensus that the CBM is responsible for the majority of non-productive adsorption to lignin. Engineering both domains of *T. reesei* Cel7A may be required to fully alleviate lignin inhibition. The highly homologous *T. emersonii* CD was not inhibited by lignin, perhaps due to this higher melting temperature of the enzyme (31, 32). Engineering the CD for greater thermal stability may therefore be a promising strategy to reduce lignin inhibition.

The wild type Te-Tr chimera, isolated *T. emersonii* CD, and 31 mutants, each with one position in the CBM mutated to alanine were produced and screened for binding to microcrystalline cellulose (Avicel) and lignin. The isolated CD had dramatically reduced affinity for lignin, consistent with previous reports that the CBM is responsible for the majority of lignin binding (17, 22).

Mutation to alanine of the three tyrosines (Tyr-5, Tyr-31, and Tyr-32) on the planar face of the CBM significantly reduced cellulose and lignin binding. These tyrosine residues are known to be essential for cellulose affinity (21), although only Tyr-32 has been evaluated previously for lignin binding (26). According to NMR data, mutation of Tyr-31 and Tyr-32 to alanine does not cause significant structural changes, and mutation of Tyr-5 to alanine causes only minor structural changes (21), indicating that the reductions in binding are due to diminished interactions between these residues and the lignin surface. The three tyrosine residues likely contribute to lignin adsorption through Pi stacking interactions with phenolic groups on the lignin surface. This result supports the hypothesis that the planar, cellulose-binding face of the CBM contributes significantly to lignin affinity.

Mutation of Gln-7 on the cellulose-binding face of the CBM also greatly reduced the binding to cellulose and lignin. This residue has been predicted to interact with cellulose through hydrogen bonding (33), although its impact on affinity had not been previously assessed experimentally. Loss of these hydrogen-bonding interactions likely causes the decrease in cellulose affinity and may also cause the reduction in lignin affinity. However, the decrease in affinity to both cellulose and lignin may also be due to structural alterations in the CBM caused by this mutation.

Mutation of an internal tyrosine (Tyr-13) to alanine also significantly decreased affinity for both cellulose and lignin. Tyr-13 has been suggested to undergo a conformational change when the CBM is bound to cellulose, moving away from the protein interior to the wedge surface to establish van der Waals contact with the cellulose chain (34, 35). Mutation of Tyr-13 to alanine may decrease affinity due to loss of these van der Waals contacts, although disruptive structural changes (caused by mutation of a large, internal hydrophobic residue) may also play a important role.

Interestingly, Q34A, which had greatly reduced cellulose affinity, retained lignin affinity similar to that of the wild type enzyme. This residue is predicted to interact with the surface of cellulose through hydrogen bonding. NMR data indicate that the Q34A mutation results in only small conformational perturbations (21). Therefore, the selective reduction in binding to cellulose implies that this hydrogen bond is important for cellulose, but not lignin, affinity. Asn-29 and Ile-11, also predicted to interact with cellulose through hydrogen bonding (35), also retained a greater fraction of their lignin affinity than their cellulose affinity. The N29A mutation has been shown to disrupt the stability of the loop containing Tyr-31 and Tyr-32 (21), and the I11A mutation has not been assessed for structural perturbations. Therefore, their role in interacting with the surface of cellulose or lignin is more difficult to ascertain. Nevertheless, these results suggest that hydrogen bonding is more important for binding to cellulose than binding to lignin. The hydrogen-bonding residues at specific positions in the CBM are able to align with the uniformly spaced hydroxyls in crystalline cellulose. In contrast, the free-radical polymerized lignin structure lacks the precise repeating structures of crystalline cellulose, making specific hydrogen bonding on the CBM surface less likely.

The remaining mutants with severely reduced affinity for cellulose and lignin are G10A, G15A, G22A, and L36A. It is unlikely that these glycine residues interact with the surface of cellulose or lignin. Gly-10 and Gly-22 are completely conserved in an alignment of homologous family one CBMs and Gly-22 is conserved as either a glycine or proline. Therefore, the decreased affinity is most likely due to structural changes caused by mutation to alanine. Mutation of Leu-36, an internal hydrophobic residue, also likely results in structural perturbations that are responsible for decreased affinity. Although these mutants had significantly reduced lignin affinity compared with the wild type chimera, they retained much greater lignin affinity than the isolated CD. This indicates that the mutated CBMs have residual lignin affinity or that the linker contributes to affinity. Linker mutations that decrease lignin inhibition are described in a patent (36), indicating that the linker does participate in non-productive adsorption to lignin.

Four mutants with increased selectivity for cellulose in the binding screen (Q2A, H4A, V18A, and P30A) were further characterized by constructing full Langmuir isotherms. Although the Langmuir model does not elucidate the complex mechanisms involved in protein adsorption to solid surfaces, the model describes the adsorption data well and is a useful tool for evaluating cellulase adsorption (37).

As expected from the binding screen, the Te-Tr wild type and mutants had similar Langmuir affinity and saturation constants for cellulose. The mutant enzymes exhibit similar lignin binding compared with the wild type enzyme at low enzyme concentration (leading to similar values for *K_A_*) but decreased binding at the higher concentrations, leading to reduced values for Γ_max_. Given the heterogeneous structure of the lignin, it is likely that there are multiple binding sites on the lignin surface with different affinities (23). Mutations in the CBM may impact interactions with one type of site more than others, resulting in decreased binding only over certain enzyme concentration ranges. The initial concentrations of enzyme employed here (up to 0.4 μmol (24 mg) Cel7A enzyme/g of cellulose) cover the range of typical enzyme loadings used in hydrolysis of lignocellulosic biomass, and therefore describe the binding behavior under industrially relevant conditions.

We next studied the impact of increased cellulose specificity on hydrolysis of Avicel in the presence of lignin and pretreated *Miscanthus*. None of the mutants outperformed the wild type enzyme, likely because lignin affinity was not reduced enough to impact hydrolysis. To test this hypothesis, we used the Langmuir model to predict the amount of each enzyme bound to cellulose under the hydrolysis conditions. Because the first step of hydrolysis is adsorption to cellulose, the calculated binding should reflect the relative hydrolytic performance of similar enzymes with different affinities. This analysis assumes that the binding to both Avicel and lignin is reversible, the amount of Avicel and lignin are constant, and that the Langmuir isotherms collected at room temperature accurately describe the binding behavior at 60 °C, the hydrolysis temperature. Although limited, this approach enables comparison of enzymes with different affinities, and estimates the magnitude of change in specificity necessary to generate an enzyme with less lignin inhibition. None of the mutants were predicted to bind in larger quantities to cellulose than the wild type enzyme, supporting the conclusion that lignin affinity must be reduced further to improve hydrolysis.

A central conclusion from the combined results of this study is that improving cellulose-hydrolyzing activity in the presence of lignin will require an even greater reduction in CBM/lignin affinity accompanied by a high level of cellulose binding. Although the mutants' cellulose and lignin affinity were highly correlated, outliers with increased specificity toward either cellulose or lignin were identified that gave insights into the mechanisms of adsorption. The four residues where mutation to alanine increased selectivity (Gln-2, His-4, Val-18, and Pro-30) are good targets for further mutation. Because hydrophobic interactions have been implicated in lignin adsorption (18, 24, 26), changing these residues to polar or charged amino acids may produce a more pronounced decrease in lignin adsorption and consequently better hydrolytic performance in the presence of lignin.

## Author Contributions

K. L. S., K. A. P., H. W. B., and D. S. C. designed the study. K. L. S. performed the experiments. All authors analyzed the results and approved the final version of the manuscript.

## Footnotes

2 The abbreviations used are:

CD: catalytic domain
CBM: carbohydrate-binding module
Te: *T. emersonii*
Tr: *T. reesei*
Te-Tr: *T. emersonii* CD with *T. reesei* linker and CBM.

## References

[1] Klein-Marcuschamer D., Oleskowicz-Popiel P., Simmons B. A., Blanch H. W. (2012) The challenge of enzyme cost in the production of lignocellulosic biofuels. Biotechnol. Bioeng. 109, 1083–1087 22095526 10.1002/bit.24370

[2] Ishizawa C. I., Jeoh T., Adney W. S., Himmel M. E., Johnson D. K., Davis M. F. (2009) Can delignification decrease cellulose digestibility in acid pretreated corn stover? Cellulose 16, 677–686

[3] Kumar L., Arantes V., Chandra R., Saddler J. (2012) The lignin present in steam pretreated softwood binds enzymes and limits cellulose accessibility. Bioresour. Technol. 103, 201–208 22047660 10.1016/j.biortech.2011.09.091

[4] Kumar R., Wyman C. E. (2009) Access of cellulase to cellulose and lignin for poplar solids produced by leading pretreatment technologies. Biotechnol. Prog. 25, 807–819 19504581 10.1002/btpr.153

[5] Ju X., Engelhard M., Zhang X. (2013) An advanced understanding of the specific effects of xylan and surface lignin contents on enzymatic hydrolysis of lignocellulosic biomass. Bioresour. Technol. 132, 137–145 23395766 10.1016/j.biortech.2013.01.049

[6] Mooney C. A., Mansfield S. D., Touhy M. G., Saddler J. N. (1998) The effect of initial pore volume and lignin content on the enzymatic hydrolysis of softwoods. Bioresour. Technol. 64, 113–119

[7] Yoshida M., Liu Y., Uchida S., Kawarada K., Ukagami Y., Ichinose H., Kaneko S., Fukuda K. (2008) Effects of cellulose crystallinity, hemicellulose, and lignin on the enzymatic hydrolysis of *Miscanthus sinensis* to monosaccharides. Biosci. Biotechnol. Biochem. 72, 805–810 18323635 10.1271/bbb.70689

[8] Rahikainen J. L., Moilanen U., Nurmi-Rantala S., Lappas A., Koivula A., Viikari L., Kruus K. (2013) Effect of temperature on lignin-derived inhibition studied with three structurally different cellobiohydrolases. Bioresour. Technol. 146, 118–125 23920120 10.1016/j.biortech.2013.07.069

[9] Rahikainen J., Mikander S., Marjamaa K., Tamminen T., Lappas A., Viikari L., Kruus K. (2011) Inhibition of enzymatic hydrolysis by residual lignins from softwood: study of enzyme binding and inactivation on lignin-rich surface. Biotechnol. Bioeng. 108, 2823–2834 21702025 10.1002/bit.23242

[10] Nakagame S., Chandra R. P., Saddler J. N. (2010) The effect of isolated lignins, obtained from a range of pretreated lignocellulosic substrates, on enzymatic hydrolysis. Biotechnol. Bioeng. 105, 871–879 19998278 10.1002/bit.22626

[11] Nakagame S., Chandra R. P., Kadla J. F., Saddler J. N. (2011) The isolation, characterization and effect of lignin isolated from steam pretreated Douglas-fir on the enzymatic hydrolysis of cellulose. Bioresour. Technol. 102, 4507–4517 21256740 10.1016/j.biortech.2010.12.082

[12] Nakagame S., Chandra R. P., Kadla J. F., Saddler J. N. (2011) Enhancing the enzymatic hydrolysis of lignocellulosic biomass by increasing the carboxylic acid content of the associated lignin. Biotechnol. Bioeng. 108, 538–548 21246506 10.1002/bit.22981

[13] Lai C., Tu M., Shi Z., Zheng K., Olmos L. G., Yu S. (2014) Contrasting effects of hardwood and softwood organosolv lignins on enzymatic hydrolysis of lignocellulose. Bioresour. Technol. 163, 320–327 24835744 10.1016/j.biortech.2014.04.065

[14] Berlin A., Balakshin M., Gilkes N., Kadla J., Maximenko V., Kubo S., Saddler J. (2006) Inhibition of cellulase, xylanase and β-glucosidase activities by softwood lignin preparations. J. Biotechnol. 125, 198–209 16621087 10.1016/j.jbiotec.2006.02.021

[15] Berlin A., Gilkes N., Kurabi A., Bura R., Tu M., Kilburn D., Saddler J. (2005) Weak lignin-binding enzymes. in Twenty-Sixth Symposium on Biotechnology for Fuels and Chemicals, pp. 163–170, Humana Press, Hoboken, NJ 10.1385/abab:121:1-3:0163 15917596

[16] Machado D. L., Neto J. M., Pradella J. G., Bonomi A., Rabelo S. C., da Costa A. C. (2014) Adsorption characteristics of cellulase and glucosidase on Avicel, pretreated sugarcane bagasse and Lignin. Biotechnol. Appl. Biochem. 10.1002/bab.1307 25322902

[17] Palonen H., Tjerneld F., Zacchi G., Tenkanen M. (2004) Adsorption of *Trichoderma reesei* CBH I and EG II and their catalytic domains on steam pretreated softwood and isolated lignin. J. Biotechnol. 107, 65–72 14687972 10.1016/j.jbiotec.2003.09.011

[18] Sammond D. W., Yarbrough J. M., Mansfield E., Bomble Y. J., Hobdey S. E., Decker S. R., Taylor L. E., Resch M. G., Bozell J. J., Himmel M. E., Vinzant T. B., Crowley M. F. (2014) Predicting enzyme adsorption to lignin films by calculating enzyme surface hydrophobicity. J. Biol. Chem. 289, 20960–20969 24876380 10.1074/jbc.M114.573642 PMC4110302

[19] Payne C. M., Knott B. C., Mayes H. B., Hansson H., Himmel M. E., Sandgren M., Ståhlberg J., Beckham G. T. (2015) Fungal cellulases. Chem. Rev. 115, 1308–1448 25629559 10.1021/cr500351c

[20] Boraston A. B., Bolam D. N., Gilbert H. J., Davies G. J. (2004) Carbohydrate-binding modules: fine-tuning polysaccharide recognition. Biochem. J. 382, 769–781 15214846 10.1042/BJ20040892 PMC1133952

[21] Linder M., Mattinen M. L., Kontteli M., Lindeberg G., Ståhlberg J., Drakenberg T., Reinikainen T., Pettersson G., Annila A. (1995) Identification of functionally important amino acids in the cellulose-binding domain of *Trichoderma reesei* cellobiohydrolase I. Protein Sci. 4, 1056–1064 7549870 10.1002/pro.5560040604 PMC2143141

[22] Rahikainen J. L., Martin-Sampedro R., Heikkinen H., Rovio S., Marjamaa K., Tamminen T., Rojas O. J., Kruus K. (2013) Inhibitory effect of lignin during cellulose bioconversion: the effect of lignin chemistry on non-productive enzyme adsorption. Bioresour. Technol. 133, 270–278 23428824 10.1016/j.biortech.2013.01.075

[23] Pfeiffer K. A., Sorek H., Roche C., Strobel K., Blanch H. W., Clark D. S. (2015) Evaluating endoglucanase Cel7B-lignin interaction mechanisms and kinetics using quartz crystal microgravimetry. Biotechnol. Bioeng. 10.1002./bit.25657 25994114

[24] Börjesson J., Engqvist M., Sipos B., Tjerneld F. (2007) Effect of poly(ethylene glycol) on enzymatic hydrolysis and adsorption of cellulase enzymes to pretreated lignocellulose. Enzyme Microb. Technol. 41, 186–195

[25] Nordwald E. M., Brunecky R., Himmel M. E., Beckham G. T., Kaar J. L. (2014) Charge engineering of cellulases improves ionic liquid tolerance and reduces lignin inhibition. Biotechnol. Bioeng. 111, 1541–1549 24522957 10.1002/bit.25216

[26] Rahikainen J. L., Evans J. D., Mikander S., Kalliola A., Puranen T., Tamminen T., Marjamaa K., Kruus K. (2013) Cellulase-lignin interactions-the role of carbohydrate-binding module and pH in non-productive binding. Enzyme Microb. Technol. 53, 315–321 24034430 10.1016/j.enzmictec.2013.07.003

[27] Shill K., Padmanabhan S., Xin Q., Prausnitz J. M., Clark D. S., Blanch H. W. (2011) Ionic liquid pretreatment of cellulosic biomass: enzymatic hydrolysis and ionic liquid recycle. Biotechnol. Bioeng. 108, 511–520 21246505 10.1002/bit.23014

[28] Labbé S., Thiele D. J. (1999) Copper ion inducible and repressible promoter systems in yeast. Methods Enzymol. 306, 145–153 10432452 10.1016/s0076-6879(99)06010-3

[29] Dana C. M., Saija P., Kal S. M., Bryan M. B., Blanch H. W., Clark D. S. (2012) Biased clique shuffling reveals stabilizing mutations in cellulase Cel7A. Biotechnol. Bioeng. 109, 2710–2719 22887329 10.1002/bit.24708

[30] Zheng L., Baumann U., Reymond J. L. (2004) An efficient one-step site-directed and site-saturation mutagenesis protocol. Nucleic Acids Res. 32, e115 15304544 10.1093/nar/gnh110 PMC514394

[31] Voutilainen S. P., Puranen T., Siika-Aho M., Lappalainen A., Alapuranen M., Kallio J., Hooman S., Viikari L., Vehmaanperä J., Koivula A. (2008) Cloning, expression, and characterization of novel thermostable family 7 cellobiohydrolases. Biotechnol. Bioeng. 101, 515–528 18512263 10.1002/bit.21940

[32] Voutilainen S. P., Murray P. G., Tuohy M. G., Koivula A. (2010) Expression of Talaromyces emersonii cellobiohydrolase Cel7A in *Saccharomyces cerevisiae* and rational mutagenesis to improve its thermostability and activity. Protein Eng. Des. Sel. 23, 69–79 19951999 10.1093/protein/gzp072

[33] Beckham G. T., Matthews J. F., Bomble Y. J., Bu L., Adney W. S., Himmel M. E., Nimlos M. R., Crowley M. F. (2010) Identification of amino acids responsible for processivity in a Family 1 carbohydrate-binding module from a fungal cellulase. J. Phys. Chem. B 114, 1447–1453 20050714 10.1021/jp908810a

[34] Nimlos M. R., Beckham G. T., Matthews J. F., Bu L., Himmel M. E., Crowley M. F. (2012) Binding preferences, surface attachment, diffusivity, and orientation of a family 1 carbohydrate-binding module on cellulose. J. Biol. Chem. 287, 20603–20612 22496371 10.1074/jbc.M112.358184 PMC3370244

[35] Alekozai E. M., GhattyVenkataKrishna P. K., Uberbacher E. C., Crowley M. F., Smith J. C., Cheng X. (2014) Simulation analysis of the cellulase Cel7A carbohydrate binding module on the surface of the cellulose Iβ. Cellulose 21, 951–971

[36] Scott B. R., St-Pierre P., Lavigne J., Masri N., White T. C., Tomashek J. J. ( 9 2, 2010) Novel lignin-resistant cellulase enzymes. U. S. Patent 20100221778 A1

[37] Nidetzky B., Steiner W., Claeyssens M. (1994) Cellulose hydrolysis by the cellulases from *Trichoderma reesei*: adsorptions of two cellobiohydrolases, two endocellulases and their core proteins on filter paper and their relation to hydrolysis. Biochem. J. 303, 817–823 7980450 10.1042/bj3030817 PMC1137620

[38] Kraulis J., Clore G. M., Nilges M., Jones T. A., Pettersson G., Knowles J., Gronenborn A. M. (1989) Determination of the three-dimensional solution structure of the C-terminal domain of cellobiohydrolase I from *Trichoderma reesei*: a study using nuclear magnetic resonance and hybrid distance geometry-dynamical simulated annealing. Biochemistry 28, 7241–7257 2554967 10.1021/bi00444a016

